# Supplementary material for: Are carbon emissions trading and green financial instruments synergistic? -Comprehensive quantitative research based on content analysis
Source: PLoS One. 2024 Mar 7;19(3):e0298601. doi: 10.1371/journal.pone.0298601 (PMC10919601; doi:10.1371/journal.pone.0298601)
Supplement: S1 Data — (ZIP) [file pone.0298601.s001.zip › Data/地级市Z_F工作报告.zip!/╣π╢1⁄2/╔ε█┌╩╨/└┤╫╘╣1⁄2╓┌║┼í╛┬φ┐╦╩2╛▌═°í┐_2018.docx]

各位代表：

　　现在，我代表深圳市人民政府向大会作政府工作报告，请予审议，并请各位政协委员和其他列席人员提出意见。

　　一、2017年工作回顾

　　过去一年，我们在以习近平同志为核心的党中央坚强领导下，认真学习贯彻习近平新时代中国特色社会主义思想和党的十九大精神，全力落实党中央国务院、省委省政府和市委的各项工作部署，坚持稳中求进工作总基调，坚持新发展理念，统筹推进稳增长、促改革、调结构、惠民生、防风险，持续打造深圳质量、深圳标准，扎实开展“城市质量提升年”工作，加快建设现代化国际化创新型城市，推动经济社会发展取得了新成绩。

　　——经济发展有速度有质量。初步核算，2017年本市生产总值2.24万亿元、同比增长8.8%；辖区公共财政收入8624亿元、增长9.2%；地方一般公共预算收入3332.1亿元、同口径增长10.1%；规模以上企业利润平均增长22.7%；居民人均可支配收入增长8.7%。

　　——社会民生事业再上新水平。新增登记就业10万人，城镇登记失业率在3%以下；新增幼儿园学位2万个、公办中小学学位4.7万个；增加10家三甲医院和三级医院，新增病床3000张、养老床位1000张；新开工及筹集人才住房和保障性住房10.2万套，建成住房租赁监管和服务平台；最低工资标准由2030元/月提高至2130元/月，最低生活保障标准由800元/月提高至900元/月；119项年度民生实事完成118项。

　　——生态环境更加优美。全市PM2.5年均浓度28微克/立方米，是全国空气质量最好的十大城市之一；建成区36条45段黑臭水体经治理基本消除黑臭；预计万元GDP能耗下降4.2%、水耗下降10.3%；建成人才公园、香蜜公园和深圳湾滨海西段休闲带等一批精品公园，“深圳蓝”“深圳绿”成为城市最亮色。

　　——城市软实力、影响力又有新提升。荣获第五届全国文明城市称号，被评为全国创新创业环境最优城市之一、全国安全感最好城市之一、全国法治政府建设典范城市；成功举办第19届国际植物学大会、高交会、文博会、国际人才交流大会、亚太社工大会、深圳国际马拉松、首届海峡两岸棒球联赛总决赛、第六届世界海洋大会、首届中欧蓝色产业合作论坛等活动。

　　一年来，我们主要做了以下工作：

　　（一）促进经济稳健发展。深化供给侧结构性改革，出台降低实体经济成本28条政策措施，全年为企业减负1369亿元。实施扩大工业有效投资26条、外贸稳增长25条等系列措施，全年完成固定资产投资5147.3亿元、增长23.8%，增速创24年来新高；社会消费品零售总额6016.2亿元、增长9.1%；进出口总额2.8万亿元、增长6.4%。产业结构持续优化，二三产比例为41.3∶58.6，形成了先进制造业、现代服务业和优势传统产业协调发展格局。制造业保持较快发展，规模以上工业增加值8087.6亿元、增长9.3%，工业投资增长27.5%，技改投资增长71.9%。ARM中国总部、空客中国创新中心等80个优质项目落地。系统优化支持金融业发展政策，建立创业创新金融服务平台，搭建地方金融风险监测预警和监管信息系统，设立首期规模50亿元的天使投资引导基金，新引进持牌金融机构36家，新增境内外上市公司41家，小微企业贷款余额增长20%，银行业不良贷款率下降至0.94%，处于全国较低水平，金融服务实体经济能力得到新提升。

　　（二）实施新一轮创新发展战略布局。全面启动创新“十大行动计划”，组织开展重大项目技术攻关，积极筹建8个重大科技基础设施，新组建诺贝尔奖科学家实验室3家、基础研究机构3家、制造业创新中心5家、海外创新中心7家，新增福田区、腾讯等3家国家级“双创”示范基地，新设立新型研发机构11家和创新载体195家，全社会研发投入超过900亿元，占GDP比重4.13%。扎实推进省“创新八大抓手”，新增国家级高新技术企业3193家，累计达1.12万家，新增全职院士12名、“孔雀计划”团队30个，全年共引进各类人才26.3万名、增长42%。出台加强知识产权保护36条，获国家科技奖15项，获中国专利金奖5项、占全国1/5，有效发明专利5年以上维持率在85%以上、居全国第一，创新能力建设取得新突破。

　　（三）深化重点领域改革。出台基础设施供给侧结构性改革方案。制定深化国有企业改革方案及配套政策，完成5家国企混改，市属国企利润增长150%。推进财政预算管理改革，以项目库为重点编早编细编实年度预算，在全国省级财政绩效考核中位居前列。深化医药卫生体制改革，组建12家基层医疗集团，进一步强化了分级诊疗，罗湖医联体建设经验获全国推广；推进公立医院药品集团采购改革，1159种药品价格平均降幅22.6%。深化政府“放管服”和强区放权改革，取消、下放175项市级行政职权。国有文艺院团、生态环保、城管综合执法、教育综合配套、地税征管体制、辅警制度等改革取得积极成效。通过深化改革，进一步优化了营商环境，新增商事主体40万户、总量增长15.2%，新增世界500强企业2家，民间投资增长22.5%。

　　（四）着力提升开放发展水平。加快前海开发开放和自贸片区建设，新推出制度创新成果111项，其中42项全国领先；新引进汇丰前海证券等港企2400多家，新孵化港澳青年创新创业团队81个，启动深港设计创意产业园建设，前海新城建设展现新面貌。深化深港合作，与香港签署推进落马洲河套地区共同发展合作备忘录，加快了莲塘/香园围等4个口岸建设改造。出台利用外资20条，实际使用外资74亿美元、增长9.9%。对外直接投资总存量超过850亿美元，居国内城市首位。拓展“一带一路”市场，开通深圳至明斯克的首条中欧班列，与沿线国家贸易额增长19.8%。新增美国夏洛特市、法国伊夫林省等3个国际友好交流城市。新开通莫斯科、米兰、墨尔本等16条国际航线，总量增长61%。

　　（五）加强城市规划建设管理。强化城市规划的战略引领作用，形成新一轮城市总体规划初步成果。加大基础设施投入，完成投资1163.5亿元、增长29.2%。开工建设轨道交通四期12、13、14号线等5条线路，同步推进三期12个在建项目，深中通道、外环高速等33个重点项目进展顺利。开通龙华有轨电车、坪山至福田高铁快捷线、深圳至中山水上巴士，打通51条断头路。建成抽水蓄能电站和公明供水调蓄工程，新建移动通信基站1.5万座。出台提升建设工程质量24条，3个项目获国家鲁班奖，5个项目获国家优质工程奖。加大城市治理攻坚，拆除消化违法建筑2380万平方米，全面开工罗湖二线插花地棚户区改造项目，新建和升级改造公厕330座。开展消防、交通、危化品等隐患排查整治，出台建设工程安全“铁10条”，全年安全生产事故死亡人数下降15.9%；加强社会治安立体化防控，110接报总警情下降24.8%，安全稳定形势持续向好。

　　（六）开展环境治理攻坚。落实中央环保督察整改任务，开展“利剑一号”环保执法专项行动，拆除饮用水水源一级保护区88栋违法建筑，排查整治7.3万家“小散乱污”企业，解决了一批市民关心的环保热点和难点问题。实施大气质量提升30条和强化措施23条，搬迁关闭63家挥发性有机物重点监管企业，新推广新能源汽车超过2万辆，实现专营公交车100%纯电动化，淘汰黄标车2.4万辆、老旧车5.4万辆，完成3座垃圾焚烧厂烟气处理和114座垃圾转运站提标改造。全面推行市、区、街道、社区四级河长制，加快推进486个治水提质项目，新增污水管网2009公里，消除排污口2780个，完成1412个小区雨污分流改造，新增优质饮用水入户小区152个，改造108个社区的供水管网，基本建成沙井污水处理厂二期、松岗水质净化厂，较好地完成了国家和省水污染治理阶段性目标考核任务。出台打造“世界著名花城”三年行动计划，新建改建59个公园，完成滨海滨河、深南大道等6个主要节点的绿化美化，建成200个花漾街区、街心花园。盐田区成为首批国家生态文明建设示范市县。

　　（七）推动社会民生事业更好发展。九大类民生支出3198亿元、增长32.5%，其中教育、医疗支出分别增长22.8%、21.3%。新增普惠性幼儿园126所，新改扩建公办中小学34所，高考一本录取率居全省第一、全国前列，深圳北理莫斯科大学、深圳技术大学（筹）招收首批本科生，教育事业有新提升。实施健康深圳行动计划，新引进高水平医学团队100个，新增执业医师2300名，建成运营孙逸仙心血管医院新院区、深圳大学总医院、萨米医疗中心，医疗卫生事业有新成绩。

　　（八）大力发展文体事业。3部作品荣获中宣部“五个一工程”奖，深圳运动员在第十三届全运会上取得8金9银11铜优异成绩，蛇口改革开放主题博物馆、海上世界文化艺术中心建成开放，获德国红点设计奖、iF设计奖共227项、居全国首位。成功举办读书月、“一带一路”国际音乐季、中国杯帆船赛、深港城市建筑双城双年展等活动。“海上看深圳”旅游项目试运营，大鹏新区获评国家级旅游业改革创新先行区。

　　（九）认真抓好精准扶贫脱贫和区域对口合作。扎实推进援疆援藏和对口扶贫协作广西百色、河池工作，积极做好对口帮扶河源、汕尾，全年共实施支援6省34县（市、区）项目233个，投入财政帮扶资金73亿元、增长17%，较好地完成了国家、省下达的年度对口扶贫合作任务。开展深圳与哈尔滨对口合作。加强深莞惠“3＋2”经济圈建设，推进深圳与河源、汕尾产业共建，66个超亿元项目落地，深汕特别合作区开发建设进入新阶段。

　　此外，信访、统计、计生、民族、宗教、对台、侨务、民防、气象、慈善、双拥、国防动员、档案、史志等工作取得新进步。

　　过去一年，我们牢固树立“四个意识”，坚持党的领导，自觉在思想上政治上行动上同以习近平同志为核心的党中央保持高度一致。我们自觉接受人大、政协监督，认真执行市人大及其常委会决议决定，定期向市人大常委会报告工作、向市政协通报情况，提请市人大审议法规议案20件，制定政府规章13项，全面清理政府规范性文件。办理人大代表建议701件、政协提案537件，注重把办理工作与当前政府工作结合起来，推动了物业管理法规修订、中小企业减负等工作，解决了“刷脸”领取养老金、“双非”儿童就读等一系列问题。注重政民互动和信息公开，推进“互联网＋政务服务”，全面推行行政审批服务事项标准化，建立行政审批统一申办平台，推出营业执照等审批结果快递服务，尽量让市民办事“少跑腿”。认真落实党风廉政建设主体责任清单，严格落实中央八项规定精神及实施细则，加大行政监察和审计监督力度，保持反腐败高压态势，严控“三公”经费，政府职能和作风进一步转变。

　　各位代表，过去一年的成绩来之不易，这是在习近平新时代中国特色社会主义思想科学指导下取得的结果，是在省委省政府和市委坚强领导下，在市人大、市政协的监督支持下，全市人民凝心聚力、团结拼搏的结果。在这里，我代表深圳市人民政府，向全市人民，向给予政府工作大力支持的人大代表和政协委员，向各民主党派、工商联、各人民团体和社会各界人士，向中央和省各部门、中央和各省区市驻深单位，向驻深部队官兵，向关心和支持深圳改革开放创新的港澳同胞、台湾同胞、海外侨胞和国际友人，表示崇高的敬意和衷心的感谢！

　　同时，我们也清醒地认识到，当前深圳正处于高位过坎、稳中求进的关键时期，我们的总体发展水平与高质量发展的要求还有不小差距，城市环境品质与国际先进城市相比还有不小差距，基本公共服务水平与群众的期待还有不小差距，政府治理能力与超大型城市快速发展的需求还有不小差距。特别是，我们的实体经济发展压力还很大，生产经营成本偏高，土地空间有限，工业增加值率与发达国家相比还有相当差距，需要构建更加先进的现代产业体系。我们的原始创新能力还不强，基础研究投入还不足，远低于东京、首尔、北京等先进城市，高等教育发展水平、创新型人才总量与创新发展的需要不相适应。我们的基本公共服务无论是质还是量都有很多短板，学前教育总体水平偏低、监管不到位，中小学优质学位紧、班额大；医疗资源总体不足，医生缺、床位紧，基层医疗服务能力不强，千人床位数仅为3.6张，低于全国5.1张的平均水平，三甲医院只有16家，远低于北上广等城市；文体设施总量较少、布局不均、服务不足。我们的住房保障和供应体系还不够完善，房价高，人才住房和保障性住房缺口较大，中低收入、“夹心层”等群体住房问题有待系统性解决。我们的生态环境治理任务还很艰巨，全市310条河流中仍有80条为黑臭水体，污水收集率不足80%；机动车保有量超过325万辆，密度大、排放多，空气质量进一步提升难度较大。我们的城市管理还比较粗放，城中村“脏乱差”和安全隐患问题突出，历史遗留违法建筑亟需依法处置和全面纳管，垃圾减量分类和收运工作推进力度还需加大，垃圾处理能力存在较大缺口，施工扰民现象不同程度存在，公共安全风险管控存在薄弱环节，非法集资等涉众金融监管还不到位。我们的公共交通服务品质仍需提升，公交出行分担率尚低于60%，远落后于国内外先进城市水平，交通出行还不够顺畅，福龙路、梅观路、南海大道、文锦北路等主干道路较为拥堵，综合交通服务和实时引导的智能化水平还不高。我们的区域发展不平衡不充分问题突出，西密东疏、南重北轻，部分区域基础设施、公建配套设施欠账较多，基层管理相对薄弱。我们的政府服务管理水平还不高，群众办事还不方便，一些政务服务没有实现跨区域、跨部门、跨层级协同办理，事中事后监管能力亟待提高；一些公职人员精气神不足，干事创业热情有所减退，不想为、不敢为、不作为、慢作为现象和腐败案件时有发生。我们要直面这些问题，采取有力措施，尽快加以改进和解决。

　　二、2018年主要工作

　　2018年是贯彻党的十九大精神的开局之年，是改革开放40周年，是决胜全面建成小康社会、实施“十三五”规划承上启下的关键一年。做好今年政府工作很重要，我们必须坚决维护以习近平同志为核心的党中央权威和集中统一领导，自觉用习近平新时代中国特色社会主义思想武装头脑、指导实践、推动工作，不折不扣贯彻落实中央、省和市委的各项决策部署，更好地服务全国全省发展大局。

　　市委六届九次全会明确了率先建设社会主义现代化先行区的目标任务，强调要做到“五个率先”、抓好“九大战略任务”，到2020年深圳将基本建成现代化国际化创新型城市，高质量全面建成小康社会；到2035年，建成可持续发展的全球创新之都，实现社会主义现代化；到本世纪中叶，建成代表社会主义现代化强国的国家经济特区，成为竞争力影响力卓著的创新引领型全球城市。同时，全会还提出2018年要重点抓好十项工作。我们要紧紧围绕市委六届九次全会确定的目标任务，聚焦富强、民主、文明、和谐、美丽，勇担新时代深圳经济特区使命，走出一条全面贯彻习近平新时代中国特色社会主义思想和党的十九大精神、体现时代特征、具有深圳特色的发展之路，努力在新时代走在最前列、在新征程勇当尖兵。

　　今年政府工作的总体要求是：以习近平新时代中国特色社会主义思想为指导，全面贯彻党的十九大和中央经济工作会议、习近平总书记“1·5”重要讲话精神，按照“三个定位、两个率先”和“四个坚持、三个支撑、两个走在前列”以及习近平总书记对深圳工作的重要批示指示要求，落实省委十二届二次、三次全会和市委六届八次、九次全会的部署，坚持稳中求进工作总基调，践行新发展理念，紧扣社会主要矛盾变化，贯彻高质量发展的要求，统筹推进“五位一体”总体布局和协调推进“四个全面”战略布局，坚持以供给侧结构性改革为主线，统筹稳增长、促改革、调结构、惠民生、防风险各项工作，大力推进改革开放，推动质量变革、效率变革、动力变革，在打好防范化解重大风险、精准脱贫、污染防治的攻坚战方面取得扎实进展，加强和改善民生，推进全面依法治市，全面从严治党，促进经济社会持续健康发展，高质量全面建设小康社会，率先建设社会主义现代化先行区。

　　做好今年的政府工作，我们要坚持把改革开放创新作为根本路径，以改革开放40周年为新起点，着力在营商环境、前海开发开放等领域推出一批标志性、引领性的改革举措，着力在创新驱动发展方面取得更大突破，努力为全国全省改革开放创新作出新探索；坚持把高质量发展作为根本要求，坚持质量第一、效益优先，率先建设全面体现新发展理念的现代化经济体系，深入推进深圳质量、深圳标准建设，着力强化标准先行、设计支撑、质量引领、品牌带动、信誉保证，全面提升产品、工程、服务、环境等各领域质量，坚定不移打造更具时代引领性的深圳品牌；坚持把增进民生福祉作为根本目的，践行以人民为中心的发展思想，着力解决人民日益增长的美好生活需要和不平衡不充分的发展之间的矛盾，从市民最关心、最紧迫的问题着手，不断提高公共服务水平，努力让市民看病更容易、就学更方便、居住更舒心，努力让发展成果更多更好惠及全体市民；坚持把良好的生态环境作为可持续发展的根本基础，牢固树立绿水青山就是金山银山的理念，高标准优化城市规划，高品质加强环境建设，推动城市净化、绿化、美化、亮化，大力治理“大城市病”，努力打造美丽中国的深圳样本，实现更高质量、更有效率、更加公平、更可持续发展。

　　今年经济社会发展的主要预期目标是：地区生产总值增长8%以上，地方一般公共预算收入增长8.5%以上，居民收入与经济增长基本同步。新增就业岗位8万个，最低生活保障标准提升至每月1000元左右，城镇登记失业率控制在3%以内，居民消费价格涨幅3%以下。生态环境持续改善，PM2.5年均浓度下降至26微克/立方米，全面完成年度节能减排任务。新开工及筹集人才住房和保障性住房8万套、供应4.6万套，新增病床2000张、养老床位1000张，新增幼儿园学位2万个、公办中小学学位5万个以上。金融等风险得到有效防控。

　　为了实现上述目标，我们要认真落实市委提出的继续推进“城市质量提升年”部署，抓创新、补短板、强质量、树品牌，重点做好以下十个方面工作：

　　（一）坚决打好“三大攻坚战”，努力在决胜高质量全面建成小康社会上取得新突破。一是打好防范化解重大风险攻坚战。落实地方金融监管责任，创新金融监管手段，全面开展各类新兴金融业态的风险排查治理，充分发挥金融风险监测预警和监管信息系统作用，加强与互联网企业合作，抓好风险预警甄别。完善市场准入和清理退出等机制，建立健全P2P网贷、小额贷款公司等监管制度，高压打击非法集资，加强从源头管控的长效机制建设，确保不发生区域性系统性金融风险。坚持全覆盖、零容忍，开展安全生产隐患排查整治，确保城市安全运行。落实国家房地产市场调控政策，促进房地产市场平稳健康发展。二是打好精准脱贫攻坚战。落实中央精准扶贫、精准脱贫部署和乡村振兴战略，继续推进援疆、援藏工作，加快喀什“一城一园一区一校一中心”建设，大力实施富民安居工程，帮助塔县、察隅县贫困人口加快脱贫，建成西藏非物质文化遗产博物馆。抓好对口广西百色、河池扶贫协作，实施一批产业共建、劳务协作等帮扶项目，开展携手奔小康行动。全面完成对口帮扶河源、汕尾323条村脱贫任务，加快深河人民医院、深汕中心医院等民生项目建设。做好对口帮扶四川甘孜、重庆巫山工作。继续加大投入，全年安排帮扶资金24亿元，确保全面完成对口帮扶任务。三是打好污染防治攻坚战。做好第二次全国污染源普查。深入推进河长制，系统治理、挂图作战，对全市约4000家重点在管企业、3万多家“小散乱污”企业等工业污染源实施严格排放监管，对全市农贸市场、汽车修配、食街、屠宰场、农业面源污染和初雨污染等实施专项治理，对5500个以上小区开展雨污分流改造，完成2350公里以上污水管网铺设，疏通运营好已建成的管网系统，新建和改造19座水质净化厂，力争实现生活污水收集处理率达到90%以上。加大水生态修复，完成62条河流黑臭水体综合治理，推动茅洲河、深圳河、观澜河、龙岗河、坪山河等主要河流水质明显改善，争取大部分河流水体达到地表水Ⅴ类水质，统筹拆违、截污、清淤、补水、岸绿、景美和道路配套，努力将全市河流打造成畅通的行洪通道、健康的生态廊道、秀美的休闲绿道、独特的文化驿道，还河流以宁静、和谐、美丽。严格保护饮用水源，逐步推广公共空间和小区住户使用直饮水。开展大气质量提升行动，实施“深圳蓝”可持续计划，淘汰老旧车5万辆，推广应用新能源汽车2万辆，完成重点工业企业挥发性有机物治理，强化扬尘管控，强力推行绿色施工，严格管治工程施工污染和餐饮油烟污染，确保空气质量位于全国城市前列。研究推进土壤污染控制。制定生态环境保护责任清单，率先开展环境污染强制责任保险试点，用最严格的制度、最刚性的执法守好绿水青山。

　　（二）深化营商环境改革，坚定不移营造更加市场化国际化法治化的营商环境。一是深入推进自贸区改革试点。积极争取国家支持，探索建设自由贸易港。深化深港金融合作，探索港资主体发起或参与设立法人银行机构、非银行金融机构，探索开展跨境人民币信贷资产转让、跨境经纪和跨境资产管理业务。争取进一步放开港澳认证机构进入自贸区开展认证检测业务。二是推进通关改革。建设智慧口岸，全面推广国际贸易“单一窗口”，支持设立全球集拼分拨中心，推进跨部门一次性联合检查，进一步压缩进出口货物通关时间。三是深化商事制度改革。全面落实准入前国民待遇加负面清单管理制度，试点将外商投资企业设立备案事项整合到商事登记“多证合一”，推动深港跨境商事登记电子化。四是推进建筑工程审批制度改革。试点企业投资项目承诺制，优化建设项目审批流程，进一步压缩审批时间，加快推动项目落地。五是着力保障产业空间。继续实施“拓展空间保障发展”十大专项行动，出台工业区块线管理办法，稳定全市工业用地总规模，严控“工改居”“工改商”，加大“工改工”支持力度，推广“工业上楼”，鼓励采取先租后让、产权入股、异地置换、厂房联建等方式，推进旧工业区连片改造升级，盘活低效用地资源。六是降低企业成本。落实国家、省税费减免政策措施，全面清理规范行业协会收费、行政审批中介收费，实现制造业用电成本下降10%，全年为企业减负1300亿元以上。七是依法实施最严格的知识产权保护。推进知识产权综合管理改革，实施知识产权重大工程项目，建设中国（深圳）知识产权保护中心、南方知识产权运营中心等平台，引进高端知识产权服务机构，完善知识产权激励、质押融资政策，构建快速受理、授权、确权、维权服务体系，高水平打造国家知识产权示范城市。八是完善社会信用体系。开展“诚信深圳”行动，搭建全市统一征信平台，全面加强信用信息的采集、共享、使用，在个人积分入户、保障房申请、公共资源交易、产业资金、政府采购等领域率先应用信用信息，推进智慧信用监管，降低制度性交易成本，实现守信联合激励、失信联合惩戒，努力打造一流信用环境。九是创新人才公共服务。制定鼓励海外人才来深创业就业政策，争取试点外籍人才多次往返签证。完善人才住房、教育、医疗、社保等服务，探索建立人才住房先租后买、以租抵购制度，高水平建设一批国际学校和国际化社区，推动建立国际医保结算服务体系，努力营造一流的人才环境。

　　（三）深化供给侧结构性改革，加快推动经济高质量发展。一是打造中国制造2025国家级示范区。坚定不移做强更具比较优势的深圳制造，大力发展高端制造、智能制造、绿色制造，培育电子信息产业、新能源、生物产业等先进制造业集群，新增3家制造业创新中心。扩大工业投资，争取完成工业投资1000亿元以上。实施技改倍增计划，完成技改投资400亿元以上。出台工业互联网行动计划和若干措施，推动2个工业互联网平台建设。开展智能制造应用示范，争创3个国家级和5个省级智能制造试点示范项目，推进一批智能制造示范工厂建设。推动黄金珠宝、眼镜、钟表、服装等优势传统产业向都市时尚产业转型，创建知名品牌示范区。二是加快重大项目落地投产。制定完善土地供给政策，加强对先进制造业、创新型企业等用地保障。实施大项目带动、产业链拓展行动，新引进超10亿元重大产业项目10个以上、总部经济项目10个以上。加快推进华星光电新型显示、亚太高通量宽带通信系统等重大项目建设，建成投产中芯国际12英寸生产线、开沃新能源汽车等项目，全年安排重大项目投资1683亿元、增长18%。三是提升现代服务业发展能级。做强做优金融支柱产业，出台支持绿色金融和金融人才发展的政策措施，大力发展金融科技、消费金融、财富管理、互联网征信等，新引进持牌金融机构25家以上，推动央行金融科技研究院等项目落地，打造市级金融控股平台，提高金融服务实体经济效能。推进国际消费中心城市建设，打造口岸经济带。壮大物流产业，重点发展现代供应链管理和航空物流，强化物流枢纽功能。提升会计、律师、咨询、设计等专业服务业水平，发展会展经济。四是激发各类市场主体活力。弘扬优秀企业家精神，充分发挥创新创业者的作用，深化国有企业改革，大力发展混合所有制企业，壮大民营经济。支持中小企业发展，设立20亿元中小微企业银行贷款风险金，组建第二批50亿元中小企业发展基金，争取新增“专精特新”中小企业60家、境内外上市企业30家以上。完善企业服务机制，建立市区两级集中统一的企业服务平台，深入开展挂点服务企业活动。

　　（四）加快建设国际科技产业创新中心，推动创新发展更有质量。坚持把创新作为引领发展的第一动力，强化产业、研发、市场、资本、人才等全要素协同，实施综合创新生态优化计划，坚定不移建设更具国际竞争力的创新之都。一是加快补齐原始创新能力短板。继续实施创新“十大行动计划”，启动大科学装置群建设，推进国家超算深圳中心二期、国家基因库二期等重大科技基础设施建设，高标准建设网络空间科学与技术省实验室和健康科学实验室等重大科研平台，新启动2家诺贝尔奖科学家实验室和2家基础研究机构建设，筹建深圳医科院，全年新增国家省市级重点实验室、工程实验室等各类创新载体100家以上，新增国家级高新技术企业2000家以上。实施重大科技项目计划，组织重大攻关项目200个以上，着力突破工业母机、5G移动通信、合成生物学等核心技术。出台支持外资研发中心发展的政策措施，新建10家以上新型研发机构。二是培育新动能。继续大力发展新兴产业，出台促进新一代人工智能、集成电路产业发展实施方案，成立人工智能产业联盟，大力推动人工智能规模化应用，规划建设石墨烯产业基地，培育第三代半导体、新材料等创新型产业。发展壮大数字经济、共享经济，推进军民融合创新发展。探索构建新技术新产品的准入、标准、认证、定价机制，加强与国际标准组织等合作，构建覆盖各领域的深圳标准体系。大力支持创新创业，新增创客空间40个、创客服务平台10个。三是集聚更多创新人才。坚定不移建设更具竞争力的人才特区，新引进全职院士10名、“孔雀计划”团队30个、海内外高层次人才1000名以上，积极培养引进高技能人才。推进深圳大学、南方科技大学、香港中文大学（深圳）建设高水平大学，加快深圳北理莫斯科大学、中山大学（深圳）、哈尔滨工业大学（深圳）等校区建设。争取深圳技术大学去筹设立，启动筹建深圳创意设计学院。四是促进科技成果加速产业化。出台金融支持科技创新若干措施，探索设立科技金融租赁和科技保险公司。制定促进创业投资发展的专项政策，发挥首期50亿元的天使投资引导基金作用，撬动更多社会资本投向初创期、种子期企业，加快建设风投创投中心城市。继续办好高交会、电子信息博览会、国际创客周、海外创新创业大赛等活动。

　　（五）落实粤港澳大湾区战略，构建全面开放新格局。以大湾区发展规划建设为契机，争取推动深圳更多项目纳入粤港澳大湾区科技创新、基础设施等专项实施方案，坚定不移推进更紧密更务实的深港澳合作，坚定不移打造更具全球影响力的海洋中心城市，积极对标国际化高标准投资贸易规则，发展更具国际竞争力的开放型经济。一是建设前海城市新中心。高水平优化提升前海发展规划，推动前海与周边区域联动发展，构建“大前海”发展格局，大力拓展对外合作新通道，推动自贸片区加快建成高水平对外开放门户枢纽和粤港澳深度合作示范区。实施前海城市新中心建设三年行动计划，开工新城立交、妈湾跨海通道、前海南山排水深隧系统等项目，完善快速到达、慢速体验的交通体系。制定新一轮全面深化改革方案，推动出台前海深港金融创新政策，设立深港科创基金，提升深港青年梦工场发展水平，新引进一批总部企业，新增港企2000家。二是打造湾区合作新亮点。与香港共同推动河套地区开发，加快深港科技创新特别合作区建设，加快推进园区土地整备和更新改造。加强深港教育医疗合作，共建高水平教育医疗机构。建成莲塘/香园围口岸，推进罗湖、皇岗、沙头角、深圳湾等口岸环境改造和功能优化。主动与澳门联手开拓葡语国家市场。积极参与广深科技创新走廊规划建设，出台深圳实施方案，构建高新区、坂雪岗科技城、大梧桐新兴产业带等多节点创新空间格局。三是构建“一带一路”交流合作新载体。拓展与“一带一路”沿线国家和地区经贸合作，完善企业“走出去”服务体系，启动总规模100亿元的丝路发展基金，推动深越海防合作区、中白物流园的建设发展。推进外贸强市建设，研究制定新一轮外贸扶持计划，支持外贸龙头企业做大做强，加快推进跨境电商综合试验区建设，积极培育壮大外贸新业态、新模式，推动出口产品提质量、创品牌，提高外贸综合竞争力。四是开拓全球海洋中心城市建设新领域。强化陆海统筹，建立健全海洋工作法规政策，编制专项规划和行动计划，推进大空港海洋新兴产业基地、中欧蓝色产业园等重大项目建设，用好海洋产业发展基金，发展壮大海洋航运、海洋生物、海工装备、海洋旅游、海事服务、海洋金融等产业。五是增创区域协同发展新优势。坚定不移构建更加均衡更有辐射力的大都市圈，深化区域合作，推动深莞惠“3＋2”经济圈深度融合发展，推动穗莞深城际线深圳段建设，加强与中山等珠江西岸地区产业协作，提升深中水上巴士服务水平。深化跨界河流、大气等污染联防联治。高起点建设深汕特别合作区，推动深汕高铁、新深汕高速等规划建设，加快市政基础设施、小漠国际物流港等项目建设，促进合作区建设上水平。落实深圳哈尔滨对口合作框架协议，深化两地产业、科技、教育等领域合作。

　　（六）全面加强城市规划建设，系统提升城市品质。一是高标准完成新一轮城市总体规划编制。坚持世界眼光、国际标准、中国特色、高点定位，围绕经济特区、粤港澳大湾区、“一带一路”交通枢纽和全球科技产业创新中心等建设，调整优化城市总体规划，突出精明增长、职住平衡、绿色低碳、智慧宜居，科学划定生态保护、产业用地、海洋水域等控制线，进一步优化城市空间布局，统筹地上地下空间综合开发利用，加强重点片区城市设计，努力营造以人为本的城市公共空间，着力打造产城融合、城海交融、人文相映的现代化城市。创新规划管理体制机制，全面推进“多规合一”。制定高标准的市政基础设施、园林绿化、景观照明技术标准和设计导则，加强对城市天际线、城市色彩、建筑立面、城市家具的规划管理，高起点规划建设前海、深圳湾、香蜜湖、大空港、大运新城等“城市新客厅”，不断提升城市的品位品质和城市能级。二是加快特区一体化进程。实施“东进、西协、南联、北拓、中优”战略，抓住国务院撤销深圳经济特区管理线的机遇，加大对特区一体化和东部等区域的投资力度。统筹提高各区规划建设和公共服务设施的标准，着力解决区域发展不平衡不协调问题。加快国际生物谷、北站商务区、光明凤凰城等17个重点区域开发，规划建设坪山高新区。三是推进基础设施建设大提速。以建设枢纽城市为目标，加快打造国际航空枢纽、国家铁路枢纽、世界级集装箱航运枢纽和区域城际轨道交通枢纽。全年安排基础设施投资1300亿元以上、增长25%，全面提升基础设施建设的质量和水平。加快规划建设机场第三跑道、新航站楼，新开5条以上国际航线。推进赣深高铁、深茂铁路深圳至江门段、深中通道等建设，强化海铁联运，促进海陆空综合交通基础设施互联互通，推进与泛珠区域共建内陆港，推动“一带一路”倡议实施和湾区经济深度融合发展。加快轨道交通三期、四期17个约300公里在建项目的建设，力争到2020年运营里程超过400公里。建成南坪三期、东部过境等快速干线，推进沙河西路等快速化改造和梅观高速等市政化改造，加快坂银通道、盐龙大道、坪盐通道、海滨大道、盐港东立交等道路建设。加快推进垃圾焚烧电厂、污泥处理厂等建设，升级改造垃圾转运站300座以上，建成清林径引水调蓄工程。新建燃气管道100公里，加快老旧供水管网改造。四是打造一流智慧城市。出台新型智慧城市建设总体规划和工作方案，实施高速宽带网络、全面感知体系等十大工程，促进技术、业务、数据融合，加快实现万物感知、万物互联、万物智能。全面完善物联网、云计算等信息网络基础设施，实现光纤入户率超过90%，启动5G试点，建成城市大数据中心和运营管理中心，建设可视化的城市空间数字平台。加快构建海陆空和地上地下的智慧化综合交通服务体系，逐步建设全市统一的智能停车系统。大力发展智慧民生，在智慧水务、智慧警务、智慧城管、智慧社区等领域打造一批示范项目，努力实现“科技让城市更美好”。五是推动城市更新。坚持政府主导、市场运作、规划先行、传承文化、综合配套、产城融合、宜居优先、分类处理、成片改造、有序更新的原则，完善城市更新政策，加快城市更新改造。坚持整体推进和微改提升协同并进，切实尽快提升城市环境，改善居住条件。以城市更新为抓手，盘活土地存量，导入先进产业，发展新兴业态，推动城市用地节约集约、创新发展。坚持违建“零容忍”，深入开展全天候巡查防控，确保违建零增长。实施违建拆除三年行动计划，出台处理历史遗留违法建筑实施办法，开展分类分步处置，力争今年拆除消化违建2000万平方米以上。

　　（七）着力加强精细化管理，提升城市治理现代化水平。对标国际先进城市，注重运用法规制度标准管理城市，构建权责明晰、服务为先、管理规范、执法严明的城市管理体系，努力让城市更有序、更安全。一是细化城市管理的法规规章。修改和完善房建、物业、地下空间、垃圾处理、食品安全、共享单车、动静态交通、外卖送货、工地围蔽、文明施工等管理办法，力求城市管理各个领域都有法可依。深化街道设计导则和道路配套、广告招牌、交通指引等标准化建设，推动各项标准规范向细处延伸、向基层拓展，满足精细化管理要求。二是开展城中村环境综合治理。制定城中村总体规划纲要和分类更新整治的配套政策，实施城中村综合治理三年计划，探索综合整治新模式，大力整治“脏乱差”问题，新建升级城中村、出租屋门禁视频系统，推进城中村物业管理全覆盖，优化出租屋和人口管理服务。加快管道燃气入户，规范瓶装气市场管理。加大消防安全治理投入，推进微型消防站全覆盖，提升消防装备水平。实施社区输配电改造工程，更新整治老旧线路，推进“三线”下地。开展排水管网“进村入户”，全面推进雨污分流。计划到2020年完成1600个城中村综合治理，今年完成600个以上，着力打造安全、干净、有序、和谐的文明新村。三是着力打造安全城市。坚持以制度管安全、用技术防风险，完善应急预案，提高防灾减灾能力，牢牢守住安全底线。加强消防、道路交通、危化品、地质灾害等安全治理，建成房屋安全隐患排查整治和公共安全风险分级管理体系，强化地铁、高层建筑、口岸、深基坑、特种设备等重点风险源管控，推进老旧电梯更新改造，开展“三合一”等场所安全专项整治，坚决防范重特大事故。深入推进平安深圳建设，完善社会治安防控体系，提升警务工作精细化、信息化、规范化水平，加强反恐防暴，强化信息安全和隐私保护，坚决打击电信诈骗、涉众型经济犯罪、“两抢一盗”等犯罪行为。四是加强社会治理创新。坚定不移推进更规范更有效的基层治理，健全完善社会治理体系，规范发展社会组织，加强专业社工和志愿者队伍建设，促进慈善公益事业健康发展，推进残疾人服务体系建设，积极构建和谐劳动关系。整合基层执法力量，强化社区网格员、楼长等动态巡查，夯实基层基础，维护社会和谐稳定。继续做好双拥、国防动员等工作。

　　（八）加快建设美丽深圳，打造更加和美宜居的城市环境。深入践行绿色发展理念，争创国家生态文明示范市，努力让城市回归自然、回归生态，率先打造人与自然和谐共生的美丽中国典范。一是实施绿化提质工程。完善绿色生态网络体系，提升园林绿化品质和公园服务功能，推进城市主干道和主要进出口景观创建。持续开展打造“世界著名花城”三年行动计划，新建20条花卉景观大道、10个花卉特色公园，新增50个花漾街区、100个街心花园。改扩建百花园、竹文化公园、前海紫荆园等60个公园，推进坝光银叶树等湿地公园建设，加快创建国家森林城市，努力实现城市在花园中、花园在城市中。二是实施美化提升工程。加强城市建筑立面整治，拆除违规户外广告，推进标识标牌等“多杆合一”，强化市政设施日常管养。着力破解城市垃圾难题，实施生活垃圾强制分类，完善分类投放、收集、运输、处理体系，推动生活垃圾分类全覆盖，今年要建成生活垃圾分类达标小区1700个；严格余泥渣土排放管理，推动建筑废弃物资源化利用，提高装配式建筑和绿色建筑发展水平。深入开展“厕所革命”，新建和升级改造公厕1000座以上。加强施工管理，推进裸露土地复绿，高标准实施施工围挡，强化占道施工的交通组织和指引服务，减少施工扰民，做到工完场清、还绿于民、还路于民。细化完善市域高快速路、城市广场和道路保洁工作标准及监管责任体系，全面提升城市保洁水平。建立市政设施、园林绿化损坏维修和更新维护的高效工作机制，落实人财物保障，确保城市设施高水平运行服务，让城市更干净、更美丽。三是实施亮化提标工程。加强城市夜景整体设计，建成深南大道等3条主干道路灯光长廊，提升福田等4个核心区灯光夜景，推广城市商业区一体化视觉系统，提升城市门户枢纽和重要节点景观品质，打造独具特色的灯光风景线。四是实施活力海岸工程。大力加强海洋环境污染治理，努力提升珠江口和深圳湾近岸海域环境，严格保护沙滩、红树林、湿地等生态资源，规划建设一批亲水平台、休闲通道和服务设施，提高近海亲海空间的可达性，丰富滨海体验，拓展海岸生活空间。高标准建设大鹏湾等特色滨海空间和旅游设施，更好展示深圳美丽海岸景观。

　　今年，我们要通过市区联动、全民参与，实施一系列环境提升工程，以更加优美的城市环境迎接改革开放40周年。

　　（九）深入实施文化强市战略，增强城市文化软实力。扎实推进“文化创新发展2020”，弘扬社会主义核心价值观，巩固提升全国文明城市创建成果，实施精品文艺创作工程，推动文化事业和文化产业蓬勃发展，率先形成展现社会主义文化繁荣兴盛的城市文明风尚，让深圳的城市空间布满创意元素、社会充满人文关怀、文明成为最温暖的底色。一是加快文化设施建设。高水平规划建设改革开放博物馆、国际交流中心、深圳湾文化设施带、新科技馆、美术馆新馆、第二图书馆等标志性设施，支持民间收藏博物馆等发展，加快国际会展中心建设。完成市属文艺院团改革，组建深圳歌舞剧院。二是大力发展文化创意产业。完善文化创意产业创新发展政策，支持原创研发，推动新媒体、资讯等产业发展，建成深港设计创意产业园，全年文化创意产业增加值增长10%以上。实施深圳设计提升行动计划，加快发展时尚设计、创意设计、工业设计，继续办好文博会、深圳设计周、深圳时装周等活动，叫响“深圳设计”城市品牌，把深圳打造成为设计师的乐园和时尚创意中心。三是完善公共文化服务体系。围绕庆祝改革开放40周年等主题举办系列文化活动，开展市民文明素养提升行动。加强大鹏所城等重点文物保护，举办“深圳非物质文化遗产周”“文化遗产日”，传承城市历史文脉。继续开展读书月、外来青工文体节、万场电影惠百姓等文化活动。四是加强基本公共体育服务体系建设。完善体育设施，改造提升深圳体育中心，新建100个以上便民利民健身场所，推动公共体育场馆免费或优惠向市民开放。办好深圳国际马拉松、中国杯帆船赛、ATP和WTA网球赛等赛事，争取更多的国际顶级赛事落户深圳。五是创建国家全域旅游示范区。建设覆盖全域旅游、可提供个性化服务的线上线下游客中心，打造中国邮轮旅游发展实验区，推进大鹏半岛旅游开发，优化提升城市形象宣传推广方案。做大旅游产业，推动旅游与文化产业融合发展。

　　（十）集中力量补齐民生短板，加快提升民生事业发展水平。要像抓经济建设一样抓民生保障，像落实发展指标一样落实民生任务，持续开展发展成果惠民行动，出台政策鼓励更多社会资本参与教育、医疗、文化、养老等供给，努力为市民提供更多更优质的公共服务，率先构建全面共建共享共同富裕的民生发展格局。全年办好九大类40项民生实事和一批民生微实事，安排教育、医疗财政支出分别增长25%和23%。一是提升教育事业发展质量。把教育事业放在优先位置，在资金、土地、人才等方面优先保障。新增新型公办幼儿园150所、普惠性幼儿园50所。加强幼儿园管理，开展幼儿园托底改薄专项行动，改善婴幼儿照护和儿童早期教育服务。新改扩建公办中小学34所，制定优质特色普通高中建设三年行动计划，出台规范集团化办学指导意见。新建20个职业教育校外实训基地。大力培养和引进名校长、名教师、名班主任，推进中小学校长职级制，加强教师队伍培训。着力解决中小学生课外负担重、择校热、大班额、午餐午休等突出问题，规范中小学教育培训机构。大力发展智慧教育，探索优质教育资源共建共享新机制，丰富网络课堂资源。二是推进健康城市建设。继续实施健康深圳行动计划，推动从治疾病向促健康转变。加强健康服务，深入实施“三名”工程，新增三甲医院2家，新引进高水平医学团队20个以上，新建名医诊疗中心3家，建成运营中山大学附属第七医院，开工建设新华医院、第二儿童医院等医疗项目。加强健康管理，大力发展智慧医疗、普惠医疗，建立健全居民电子健康档案，做强社康中心，做实家庭医生服务，9类重点人群签约服务覆盖率提高至65%以上。完善健康保障，深化医保支付方式改革，扩大医保“总额管理、结余留用”实施范围，拓展医保异地就医直接结算，鼓励社会力量发展商业健康保险和医保经办服务。发展健康产业，推动生物产业基地等建设，加快生物医药、医疗器械、精准医疗等产业发展，推进医养融合，发展养老服务业。倡导健康生活，普及健康教育，实施青少年等重点人群口腔综合防治项目，广泛开展合理饮食、适量运动、心理平衡的健康宣传教育。营造健康环境，将健康理念融入城市规划、建设和管理，建设一批健康步道、休闲广场。推进市社会救助安置院、市社会福利院、第二强制戒毒所等民政、政法设施建设，积极创建无障碍城市。三是加大住房供应和保障力度。出台新一轮住房制度改革实施意见和配套文件，着力破解中低收入、“夹心层”、专业人才等群体住房难问题，制定面向2035年的住房供应和保障计划，加快建立多主体供给、多渠道保障、租购并举的住房制度。推进光明长圳、坪山竹坑等一批人才住房和保障性住房项目建设。探索TOD模式，在轨道交通沿线筹建大型安居社区。大力发展住房租赁市场，发挥在线运行的住房租赁监管和服务平台作用，让市民阳光租房、智慧租房。出台棚户区改造政策，推进华富村等一批老旧小区重建。四是推出一批交通便民措施。坚持公交优先，大力发展以轨道交通为骨干的公共交通，注重做好地面、地下交通的接驳服务，深入开展交通拥堵大整治。新开和优化调整公交线路85条以上、微巴线路10条以上，优化地铁、有轨电车和公共巴士、出租车的运营服务，努力实现公交分担率提高至60%以上。治理交通拥堵节点100个，打通断头路20条，建成智慧立体停车库20个。大力发展智慧交通，提高交通组织效率，实现公交、地铁移动支付全覆盖。完善城市慢行系统，建成自行车专用道200公里以上，推广一批共享单车智能停放点。大力整治交通秩序，全面整顿电动单车逆线行驶等违规违章行为。五是努力打造市民满意的食品安全城市。落实“四个最严”要求，实施食品安全战略，建立“供深食品”标准体系，推进农贸市场升级改造，推广快检车、快检室，大力实施阳光智慧餐饮工程，加强网上订餐等新业态监管。建立健全全链条可追溯监管体系，从源头上保障食品安全，积极创建国家食品安全示范城市。

　　做好政府工作，需要不断加强自身能力建设。政府将牢记全心全意为人民服务的宗旨，把市民满不满意作为衡量政府工作的根本标准，坚持崇尚实干、追求卓越，进一步增强政府公信力和执行力，努力建设人民满意的服务型政府。一是抓学习、深调研、重实干。按照市委的部署要求，把深入学习宣传贯彻习近平新时代中国特色社会主义思想和党的十九大精神作为首要政治任务，牢固树立“四个意识”，坚定“四个自信”，开展“不忘初心、牢记使命”主题教育和“大学习、深调研、真落实”活动，以严实作风推动政府工作全面上水平。二是提高依法行政水平，建设法治政府。认真落实法治中国示范城市建设实施纲要，坚决执行市人大及其常委会各项决议决定，自觉接受市人大及其常委会的法律监督、工作监督和市政协的民主监督，坚持重大事项向市人大报告、向市政协通报，认真办好人大代表建议和政协提案。严格落实重大行政决策合法性审查制度。推进行政执法全过程记录，建设全市统一的行政执法监督网络平台。三是加强政民互动，建设阳光政府。加大财政预决算、住房保障等重点领域信息公开力度，公布财政专项资金、人才公共服务等8张清单。坚持问需于民、问政于民，积极回应市民关切，自觉接受新闻媒体和社会监督。四是深入推进“放管服”改革，建设效率政府。进一步精简各类行政审批事项，加强事中事后监管，创新监管方式，提高监管效能。建成统一的智慧政务服务平台，推进个人和企业信息一次录入、信息共享、全市通办，全面推广网上申报审批，努力实现“企业办事不出区、个人办事不出街”。五是转变工作作风，建设廉洁政府。加强政府系统党的建设，严格落实党风廉政建设主体责任，深入落实中央八项规定精神及实施细则，巩固“四风”整治成果，努力打造忠诚、干净、担当的高素质专业化公务员队伍。坚持以案治本、建章立制，完善工程招投标、城市更新等领域廉政风险防控制度，加强廉洁文化建设。强化财政绩效评价，规范专项资金管理，提高财政资金使用效益。深入开展调查研究，狠抓工作落实，强化督查考核，确保中央、省、市各项决策部署落到实处、早见实效。

　　各位代表，市民对美好生活的向往就是我们的奋斗目标。让我们更加紧密地团结在以习近平同志为核心的党中央周围，高举习近平新时代中国特色社会主义思想伟大旗帜，在市委的坚强领导下，不忘初心、牢记使命，以永不懈怠的精神状态和一往无前的奋斗姿态，率先建设社会主义现代化先行区，努力在新时代展现新作为、创造新业绩，让深圳这座年轻的城市更有活力、更具魅力，为实现中华民族伟大复兴的中国梦作出新的更大贡献！
